# Advances in chemoprevention of familial adenomatous polyposis

**DOI:** 10.3389/fonc.2026.1815387

**Published:** 2026-05-19

**Authors:** Ruicheng Li, Rong Cao, Jiaqi Kang, Yuwei Li, Xin Lin, Zhao Zhang

**Affiliations:** 1 School of Medicine, Nankai University, Tianjin, China; 2 Department of Colorectal Surgery, Tianjin Union Medical Center, The First Affiliated Hospital of Nankai University, Tianjin, China; 3 Tianjin Institute of Coloproctology, Tianjin, China

**Keywords:** APC gene, chemoprevention, familial adenomatous polyposis, nonsteroidal anti-inflammatory drugs, Wnt/β-catenin signaling pathway

## Abstract

Familial adenomatous polyposis (FAP) is an autosomal dominant hereditary disease characterized by the early onset of numerous intestinal polyps, which, if untreated, may progress to colon cancer. Traditional treatments have primarily relied on surgical resection; however, with advancements in molecular biology and pharmacology, chemoprevention has increasingly become an important adjunct in the treatment of FAP. This review systematically summarizes the molecular pathological mechanisms of FAP and recent advances in chemopreventive strategies, including nonsteroidal anti-inflammatory drugs (NSAIDs) and combination regimens, prostaglandin E_2_ receptor antagonists, phytoestrogens, targeted pathway inhibitors, as well as emerging interventions including gut microbiota modulation and natural products.

## 1 Introduction

Familial adenomatous polyposis (FAP) is typically characterized by the widespread presence of hundreds to thousands of tubular or villous polyps, usually 2–10mm in diameter, which occur throughout the colon and rectum (1). Additionally, patients have an increased risk of developing other tumors, including duodenal, gastric, and thyroid cancers, as well as desmoid tumors (2). Classified according to polyp burden and the age of onset, FAP may be divided into the classical familial adenomatous polyposis (CFAP) and the attenuated familial adenomatous polyposis (AFAP) (3). CFAP is further categorized into intermediate and dense types based on the quantity of polyps (100–1000 and >1000, respectively) and is characterized by early onset and a nearly 100% cancer conversion rate. AFAP is a milder form of FAP characterized by later onset, which has less than a hundred colonic adenomas (4).

Although CFAP arises from pathogenic mutations in the adenomatous polyposis coli (APC) gene with autosomal dominant inheritance, up to one-third of adenomatous polyposis patients lack a definitive genetic diagnosis, and most are presumed non-genetic (5). Additionally, around 20% of FAP cases are induced by mutations in other genes, most notably the MUTYH-associated polyposis (MAP) associated with biallelic mutations in the MUTYH gene, which follows an autosomal recessive inheritance pattern (2, 6, 7). The overall penetrance of FAP is between 70-95%, with significant variability in phenotypic expression. No conclusive reports have identified modifying genes that affect disease penetrance or expression.

In recent years, chemopreventive strategies have advanced significantly through deeper insights into the molecular mechanisms of FAP. Daca-Álvarez et al. systematically reviewed the latest developments in non-surgical management, emphasizing personalized approaches that integrate endoscopic surveillance with chemoprevention (8). This article systematically reviews the clinical trial data on chemopreventive agents (e. g., COX-2 inhibitors), targeted therapies (e. g., Wnt/β-catenin/EGFR/PI3K/mTOR pathway inhibitors) and microbiome modulation, exploring the translational potential of multidisciplinary treatment strategies.

## 2 Molecular pathology of FAP and therapeutic needs

### 2.1 APC gene inactivation and abnormal Wnt/β-catenin signaling

**Figure 1:**
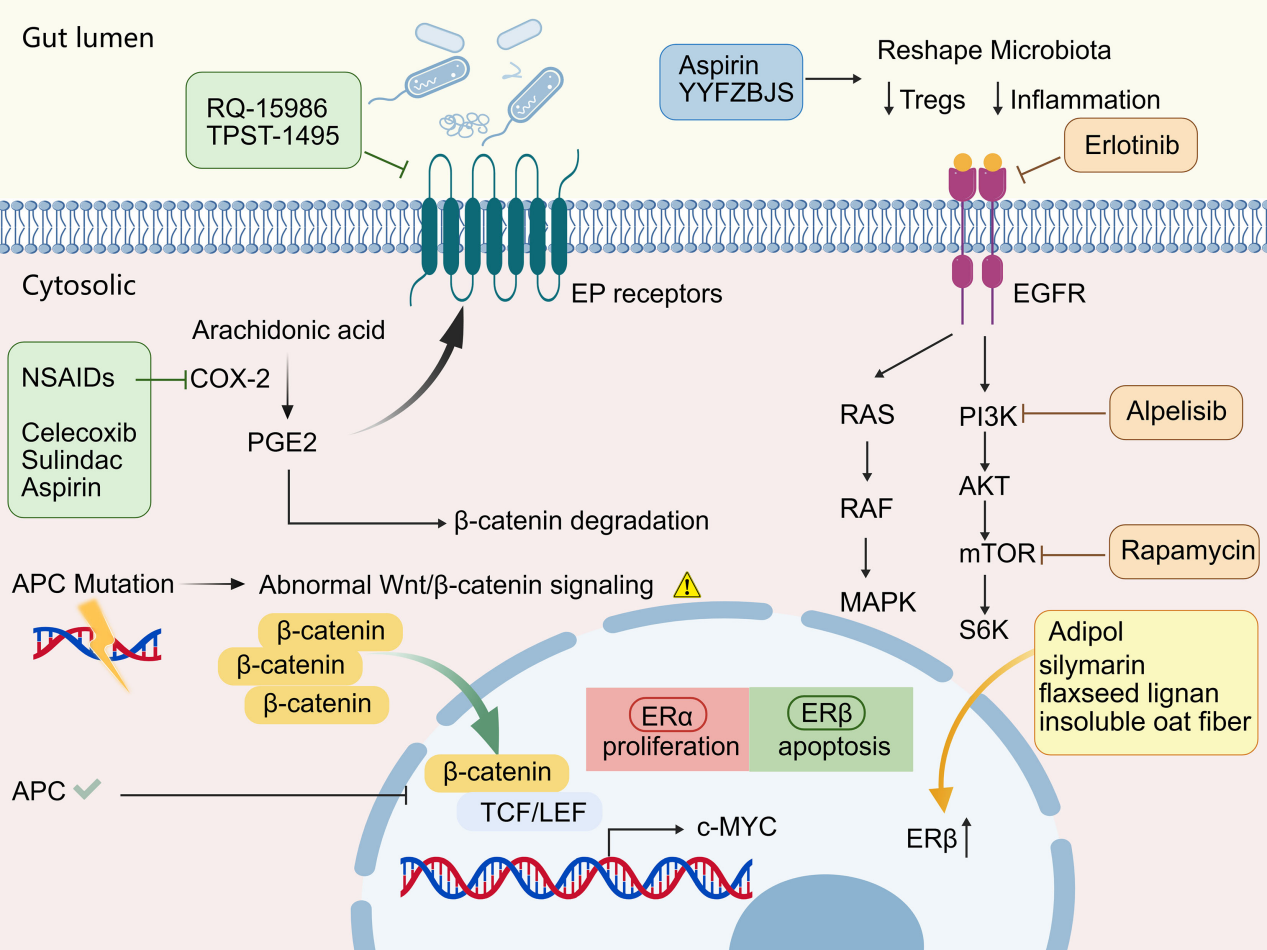
Overview of molecular pathways and targeted chemopreventive strategies in familial adenomatous polyposis (FAP).

As noted, FAP is principally induced by germline mutations in the APC gene, while its core pathological mechanism is the abnormal activation of the Wnt/β-catenin signaling pathway, resulting in nuclear accumulation of β-catenin, which drives the proliferation of intestinal epithelial cells (6, 9), ultimately causing the formation of numerous colorectal adenomas, with an extremely high risk of malignant transformation (almost 100% in untreated individuals) (10) (**Figure 1**). Additionally, somatic mutations in abnormal proliferating stem cells contribute to the carcinogenesis process, playing a significant role in the disease progression (6, 11).

### 2.2 Clinical treatment challenges

Traditionally, FAP treatment has relied heavily on surgical intervention, including total colectomy or periodic polyp resection. With the development of laparoscopic and robot-assisted laparoscopic techniques, postoperative complications have decreased, effectively reducing the risk of colorectal cancer (12). In FAP patients, the lifetime risk of duodenal adenocarcinoma approaches 20%, currently the leading cause of death, while the risk of duodenal surgery closely parallels the natural progression of duodenal adenomatosis over time (4, 13, 14). A recent study confirmed that FAP patients exhibit a 14-fold elevated risk of developing duodenal/small bowel cancer compared to the general population (15). Moreover, periodic polyp resections may only remove a limited number of larger polyps at a time, while smaller polyps are often difficult to eliminate completely.

### 2.3 Overview of chemopreventive mechanisms and targets

To comprehensively illustrate the multiple chemopreventive strategies for FAP, **Figure 1** summarizes the core molecular pathogenesis and the corresponding therapeutic targets discussed in this review. As previously noted, APC gene mutations drive the abnormal activation of the Wnt/β-catenin signaling pathway. Various agents have been shown to disrupt this and related auxiliary pathways. For instance, NSAIDs (such as celecoxib, sulindac, and aspirin) inhibit COX-2, thereby reducing prostaglandin E2 (PGE2) production and promoting β-catenin degradation. Furthermore, agents like RQ-15986 can directly antagonize EP receptors. In the realm of targeted therapies, Erlotinib, Alpelisib, and Rapamycin block the EGFR, PI3K pathways. Additionally, phytoestrogens and related nutraceutical formulations (e.g., Adipol, silymarin, flaxseed lignans) induce apoptosis by up-regulating ER-β expression. Beyond direct intracellular signaling, systemic interventions using Aspirin and traditional Chinese medicine formulas like YYFZBJS also help reshape the gut microbiota.

## 3 Nonsteroidal anti-inflammatory drugs and combination therapies

### 3.1 Traditional NSAIDs

It has been reported that NSAIDs are linked to a reduced risk of colorectal cancer (CRC), primarily by inhibiting cyclooxygenase-2 (COX-2) enzymes, reducing PGE2 production, and promoting the degradation of β-catenin (16) (**Figure 1**). Research indicates that regular use of NSAIDs like sulindac and celecoxib may slow the progression of adenomas and delay malignant transformation in FAP patients (17–20). In a 4-year trial, twice-daily weight-adjusted sulindac (75 mg for 20–44 kg, 150 mg for >44 kg) reduced colorectal polyps by 44%–64% (19), though gastrointestinal side effects such as bleeding have limited its use. Celecoxib has been reported to reduce the polyposis burden in the duodenum by 31% in the group receiving 100 mg of celecoxib twice daily, though it also poses significant cardiovascular risks (20).

### 3.2 New discoveries with NSAIDs

The recent clinical trials and outcomes of NSAIDs and combination therapies for the chemoprevention of FAP are summarized in **Table 1**. Two large prospective cohort studies showed a significant association between regular aspirin use (2 or more standard-dose tablets per week or 6 or more low-dose (81-mg) tablets per week) and an absolute reduction in the incidence of colon cancer among participants with unhealthy lifestyles (21). Fletcher et al. (22) found that NSAIDs selectively kill newly emerging tumor cells by activating death receptor signaling and promoting endoplasmic reticulum stress, which enhances tumor suppression and reduces CRC risk. Additionally, Daca-Álvarez M, et al. (8) found that sulindac at daily doses of 150–300 mg effectively reduces the size and number of colorectal adenomas, while the use of sulindac suppositories in 15 postoperative patients achieved a 90% complete adenoma regression rate with no recurrence during 33 months of follow-up. The phase II clinical study (NCT00503035) has shown that celecoxib may inhibit CRC progression by upregulating 15-lipoxygenase-1 expression, increasing its metabolites, such as 13-HODE colonic tissue levels in colorectal normal and polyp tissues (18). Aspirin, a pioneer NSAID, has also been rediscovered in a phase II, III clinical trial (UMIN000018736) involving FAP patients without previous colectomy. By evaluating the incidence of recurrence/development of colorectal polyps larger than 5.0 mm in diameter, Ishikawa et al. found that low-dose regimens (100mg/d) significantly reduced rectal polyp density in FAP patients (23). A recent meta-analysis further confirmed the efficacy of nonselective COX inhibitors in FAP, demonstrating a marked reduction in polyp burden with a safety profile comparable to placebo (24). Further research indicates that aspirin induces AMPK activation and inhibits c-MYC, NRF2/ARE/miR-34a/B signaling pathways to suppress colorectal cancer metastasis (17).

**Table 1:** Summary of recent clinical trials of NSAIDs and combination therapies.

Intervention	NCT identifiers	Trial phases	Patients	Primary outcome measures	Results	Ref.
Celecoxib	NCT00503035	Phase II	Patients with FAP	13-HODE Colonic Tissue Levels	Inhibited CRC progression by upregulating 15-lipoxygenase-1 expression, increasing its metabolites.	(18) 2022
Aspirin	NA	NA	Participants with unhealthy lifestyles	10-year cumulative incidence of CRC, absolute risk reduction (ARR), number needed to treat and hazard ratios for incident CRC.	Regular aspirin use was significantly associated with an absolute reduction in colon cancer incidence.	(21) 2024
Aspirin	UMIN000018736	Phase II,III	Patients with FAP without previous colectomy	Recurrence/development of colorectal polyps	Low-dose aspirin (100 mg/d) significantly reduced rectal polyp density in patients with FAP.	(23) 2021
Sulindac and difluoromethylornithine	NCT01483144	Phase III	Patients with FAP	Number of Subjects With Any FAP-related Event.	Extended the time to the first lower digestive tract surgery and reduced the risk of high-grade dysplasia.	(27) 2022
Sulindac and erlotinib	NCT01187901	Phase II	Patients with FAP	Change in Duodenal Polyp Burden From Baseline to 6 Months	Showed a significant reduction in polyp burden (−32.0 to −10.9).	(29) 2016

Examining through the lens of intestinal microecology, the interplay between aspirin and gut microbiota could represent a crucial pathway for preventing colorectal cancer. Research indicates that Lactobacillus is capable of breaking down aspirin, which diminishes its chemoprotective benefits in mouse models. Furthermore, aspirin consumption triggered shifts in the microbial community structure of mice, leading to elevated levels of beneficial bacteria alongside a reduction in harmful strains (25). Additionally, Yang et al. demonstrated that by modulating gut microbiota, aspirin could influence TIGIT expression on Treg cells. This modulation weakens the immunosuppressive activity of Tregs, thereby boosting the capacity of other immune cells to combat tumors within the microenvironment (26) (**Figure 1**).

In combination therapies, sulindac and difluoromethylornithine (DFMO) have shown significant improvements over monotherapy, in a phase III trial (NCT01483144) evaluating FAP-related events, notably extending the time to the first lower digestive tract surgery and reducing the risk of high-grade dysplasia (27). Moreover, combining sulindac with bexarotene (each 7.5 mg/kg/day) enhances sulindac’s inhibition of the Wnt signaling pathway and reduces polyp numbers in Apc-mutant mice, with transcriptional profiling revealing synergistic inhibition of pathways such as PI3K/AKT and MAPK (16). A recent study has demonstrated that, the combination of sulindac (30 mg/kg, intraperitoneal injection) and pyrvinium pamoate (25 mg/kg, oral gavage) administered 10 times over 14 days reduced colonic adenoma burden by 72% (p<0.01) in Apc-mutant mice, mediated through enhanced CD3+ T cell infiltration and suppression of β-catenin/TCF transcriptional activity (28). In a phase II trial (NCT01187901), Samadder et al. randomized participants with FAP to receive either sulindac (150 mg twice daily) combined with erlotinib (75 mg daily) (n=46) or placebo (n=46) for 6 months. The study found that the combination therapy remarkably decreased the polyp burden, specifically measured as the change in polyp burden from a 10-centimeter segment of the duodenum (−32.0 to −10.9), though adverse effects such as acneiform rash, oral mucositis, and diarrhea were commonly observed, potentially limiting its clinical applicability (29).

## 4 Prostaglandin E2 receptor antagonists

Prostaglandins, particularly PGE2, are key inflammatory mediators produced by COX-2 from arachidonic acid. PGE2 signaling via its receptors (EP1, EP2, EP3, EP4) profoundly influences various inflammatory processes and is closely linked to colorectal tumorigenesis. Therefore, receptor antagonists may be beneficial for treating colorectal cancers (**Figure 1**). PGE2 receptor antagonists such as RQ-15986 inhibit inflammation-associated colorectal tumors in APC-mutant rats, similar to the effects of COX-2 inhibitors (30). Notably, the novel dual EP2/EP4 receptor antagonist TPST-1495 is currently being evaluated in a Phase II clinical trial (NCT06557733) for its ability to reduce intestinal polyp burden in patients with FAP, highlighting its promising clinical translation for colorectal cancer prevention via targeted PGE2 blockade.

## 5 The action of phytoestrogens

17β-estradiol (E2) exerts its effects by binding to ERα and ERβ, two nuclear estrogen receptors. After dimerization and nuclear translocation, they induce target gene transcription. Both receptors are present in normal colorectal mucosa, with ERβ dominating. These two isoforms have opposing actions on cell replication and survival. The E2-ERα complex promotes cell survival and anti-apoptotic effects, whereas the E2-ERβ complex drives apoptosis (**Figure 1**). Recent studies have shown the effects of phytoestrogens such as silymarin and coumestrol; these plant-derived molecules with estrogenic-like features are capable of binding to estrogen receptors (6, 31). In 40 Apc^Min/+^ mice fed standard or enriched diet (20 each) for 110 days, research suggested that an enriched formulation of silymarin, boswellic acid, and curcumin exerted a synergistic chemo-preventive effect in inherited intestinal carcinoma, possibly mediated by reduced epithelial proliferation, increased apoptosis, and accelerated villous cell renewal (32). Research indicated that Adipol (a patented nutraceutical formulation composed of silymarin, flaxseed lignans, and insoluble oat fibers), established as a chemopreventive agent for FAP and CRC, reduces carcinogenesis, up-regulates ER-β expression, induces apoptosis, and is safe and well-tolerated (31, 33) (**Figure 1**). A study using Apc^Min/+^ mice found that coumestrol, a phytoestrogen found in bean sprouts, effectively suppresses the development of intestinal polyps, cancer cachexia, and hypogonadism (34).

## 6 Targeted therapy: from molecular mechanisms to clinical translation

### 6.1 Wnt/β-catenin signaling pathway interventions

The Wnt/β-catenin pathway exerts a significant impact on tissue homeostasis and is essential for stem cell differentiation, development, and self-renewal. Malignant activation of this pathway is associated with various cancers, particularly those involving APC mutations (6, 9, 16, 28, 35). Sulindac and bexarotene (each 7.5 mg/kg/day) synergistically inhibit the Wnt pathway, reducing polyp numbers in Apc-mutant mice (16). Additionally, pyrvinium pamoate, a Wnt pathway antagonist, enhances T-cell infiltration and β-catenin/TCF transcriptional activity inhibition to reduce adenoma formation in Apc-mutant mice (28). Emerging small molecules such as C644–0303 and KYA1797K show promise in blocking TCF/LEF transcriptional activity and inducing apoptosis in PDX models (35, 36). ZKN-0013 promotes readthrough of nonsense mutations in the APC gene, restoring functional APC protein and thereby suppressing the Wnt/β-catenin pathway. *In vitro*, it reduces nuclear β-catenin and c-myc levels in colon cancer cells, and *in vivo*, it decreases intestinal polyps, alleviates anemia, improves survival, and induces differentiation in Apc^Min/+^ mice (37).

### 6.2 EGFR signaling pathway intervention

APC mutations lead to persistent activation of the Wnt/β-catenin pathway, while the EGFR signaling pathway exerts a significant impact on the formation of FAP adenomas (38). The absence of EGFR pathway-related genes (e. g., ERBB3) exerts a profound genetic background-dependent effect on tumorigenesis, likely by disrupting its unique link to the PI3K/AKT and MAPK pathways and by abolishing EGFR/ERBB3, ERBB2/3, and ERBB3/4 heterodimers, thereby influencing polyp size (39).

A multicenter phase II clinical trial evaluated the efficacy and safety of Erlotinib (an EGFR inhibitor) in patients with FAP. Participants self-administered 350 mg erlotinib orally once weekly at approximately the same day/time for 6 months during the intervention phase. Among the 42 patients who completed the study, the total diameter of duodenal polyps decreased by 29.6% (p<0.0001), the quantity of polyps decreased by 10.7% (p=0.0002), and 12% of patients experienced a reduction in Spigelman staging. The number of lower gastrointestinal polyps decreased by 30.8%. The study concluded that once-weekly erlotinib remarkably reduces duodenal polyp burden in FAP patients and provides a promising chemopreventive strategy to postpone the requirement for surgery (40). Additionally, the combination of Erlotinib and Sulindac significantly reduced polyp load (29), although both studies reported side effects such as acne-like rashes and diarrhea, suggesting a need for further research to optimize dosing (29, 40).

### 6.3 PI3K/AKT/mTOR pathway intervention

Research has shown that the inactivation of the APC gene, while activating the Wnt/β-catenin pathway, also upregulates the PI3K/AKT/mTOR signaling pathway (**Figure 1**), driving glycolysis (Warburg effect) and promoting the proliferation and survival of adenoma cells (36). Moreover, in the context of APC mutations, the p110β subunit of PI3K dominates in adenomatous organoids, suggesting that PI3K pathway activation is a critical step in carcinogenesis (38). AKT acts as a direct upstream regulator of β-catenin, triggering its activation and nuclear shuttling through direct phosphorylation. Furthermore, evidence suggests that the Wnt/β-catenin axis reciprocally dictates AKT expression and activation levels, a synergy that is particularly pronounced in the microenvironment of CRC (41).

In animal experiments with Apc^Min/+^ mice, PI3K inhibitors (e. g., Alpelisib) remarkably lowered the number and volume of polyps (by 68%) (36). Rapamycin may inhibit the abnormal protein synthesis induced by APC deficiency by inhibiting the mTORC1 signaling pathway (especially the S6K-eEF2K axis), leading to differentiation of the Wnt-overexpressing progenitor cells in tumors into non-proliferative Paneth-like cells, thus inhibiting tumor initiation. Recent research indicated that Rapamycin treatment restored differentiation potential in FAP1 organoids but not in FAP2 organoids (42). Liu et al. demonstrated the combination of rapamycin/liposomes (2 mg/kg, tail vein injection twice a week) and 5-FU (30 mg/kg, intraperitoneal injection twice a week) dramatically decreased the number and load of tumors in Apc^Min/+^ mice (43). A clinical trial (NCT03095703) further showed that daily administration of rapamycin (2 mg for 6 months) decreased polyp burden via pro−apoptotic and anti−proliferative mechanisms, albeit with mild−to−moderate toxicities (44). Metformin has garnered attention for its mTOR-inhibiting properties. However, a completed 7-month randomized, double-blind, placebo-controlled trial (NCT01725490) revealed that metformin did not significantly reduce the mean number or size of colorectal and duodenal polyps in FAP patients, highlighting the complexities of translating preclinical success into human efficacy (45).

## 7 Other studies

In terms of gut microbiota and immune regulation, Sui et al. found that YYFZBJS, a traditional Chinese medicine formula, improved the progression of colorectal cancer in Apc^Min/+^ mice by increasing Lactobacillus and reducing Ruminococcaceae, reshaping the gut microbiota, downregulating the proportion of regulatory T cells (Tregs, CD4+CD25+Foxp3+ reduced by 50%), and inhibiting β-catenin phosphorylation (46) (**Figure 1**).

Regarding polyamine metabolism and epigenetic regulation, 5’-methylthioadenosine (MTA), a substrate of 5’-methylthioadenosine phosphorylase (MTAP) involved in polyamine synthesis, is converted by MTAP into S-adenosyl-L-methionine (SAM) via the salvage pathway. Research has shown that Methylthio-DADMe-Immucillin-A (MTDIA), an oral transition-state MTAP inhibitor, increases MTA levels, inhibits PRMT5-mediated methylation, improves anemia symptoms in Apc^Min/+^ mice, limits tumor growth, and extends the lifespan of these mice (47).

Regarding lipid metabolism, epidemiological studies have suggested that consuming fish oil rich in n-3 polyunsaturated fatty acids (PUFAs) may reduce the risk of colorectal cancer by modulating the inflammatory state of cell membranes. Experimental studies have revealed the function of n-3 PUFAs in colon cancer, demonstrating growth inhibition effects on malignant tumor progression. Dietary n-3 PUFAs have been shown to lower tumor burden in APC-deficient mice, possibly through certain unsaturated fatty acid derivatives that modulate the COX-2 pathway or upregulate ERβ/LDL-R and reduce FAS activity (48–50). A network meta-analysis further provided clinical evidence supporting the efficacy of n-3 PUFAs, demonstrating that eicosapentaenoic acid (EPA-FFA) at a dose of 2 g/d was the most effective intervention for reducing colorectal polyp diameter (51). Recent evidence indicates that short-chain fatty acids, particularly butyrate, protect against colorectal cancer and precancerous lesions, supporting fiber-based chemoprevention in high-risk populations such as familial adenomatous polyposis (52). The farnesoid X receptor (FXR) agonist obeticholic acid (OCA), which mimics naturally occurring bile acids, is now being investigated in a Phase IIa clinical trial (NCT05223036) for its safety and efficacy in reducing intestinal polyp burden.

Active ingredients derived from natural products have also demonstrated intervention potential. Lee et al. (53) found that *in vitro*, high-phenolic sorghum bran extract downregulated the activity and expression of β-catenin transcription in human colon cancer cells, inhibited IGF-1-induced PI3K/AKT pathway stimulation, activated AMPK, and induced autophagy in colon cancer cells to inhibit cell proliferation and trigger apoptosis. These effects were confirmed *in vivo*, where it suppressed colon tumor formation in Apc^Min/+^ mice. Furthermore, Curcumin, a natural polyphenolic compound derived from turmeric, has anti-inflammatory and antioxidant properties. A recent review indicated that curcumin may exert antitumor effects by inhibiting polyamine synthesis (54). Preclinical reports have indicated that curcumin lowered the number and volume of polyps in FAP mice, but this human study (NCT00641147) has yielded conflicting results, possibly due to insufficient blood concentration (55). Gilad et al. (56) used a turmeric-based nutritional supplement to address this issue, receiving positive feedback, with significant reductions in tumor burden and inflammatory levels in FAP patients. Based on the extended follow-up of a randomized, double-blind, placebo-controlled trial, berberine (0.3 g twice daily) was administered for two years, after which patients were observed off treatment for six years. Tan et al. found that, the group receiving berberine demonstrated a 17.4% reduction in adenoma recurrence rate and a 7.6% decrease in the incidence of neoplasia compared with the placebo group. These findings indicated that a two-year course of berberine provides sustained chemopreventive effects against colorectal adenoma recurrence and neoplasm development for at least six years after treatment cessation (57).

Research targeting specific kinases and signaling interactions has provided new avenues. HASPIN, a serine/threonine kinase specifically expressed in haploid germ cells, regulates mitotic and meiotic chromosome and spindle function by phosphorylating histone H3 serine. Tanaka et al. (58) used the HASPIN inhibitor CHR-6494 in Apc^Min/+^ mice, observing a significant reduction in small intestine polyp numbers and suppression of cachexia and gonadal dysfunction in the mice. Additionally, in colorectal cancer, APC directly interacts with Rho guanine nucleotide exchange factor 4 (Asef) to release its small GTPase activity. Activated Asef promotes abnormal migration and invasion of colorectal cancer cells through the CDC42 pathway. Yang et al. (59) showed that inhibition of APC-Asef interaction by blocking the ARM domain with Asef-SH3 could inhibit cell migration in metastatic colorectal cancer models.

Additional mechanisms had also been explored. Clarke et al. found that (60), although butyrylated high-amylose maize starch (HAMSB) ingestion had no significant effect on the primary endpoint of global polyp number, there was a trend toward a reduction in small polyps, and carryover effects were observed. Therefore, butyrate delivered by HAMSB may still hold chemopreventive potential, warranting further investigation in larger cohorts to validate its efficacy in reducing colorectal cancer risk in individuals with FAP and the general population.

## 8 Summary and prospective

Although significant progress has been made in drug treatment for FAP, many challenges remain. Traditional drugs such as NSAIDs have shown good clinical efficacy, but side effects continue to be a concern. Research into Wnt signaling inhibitors and new targeted inhibitors is promising, potentially providing more treatment options for FAP patients in the future. Meanwhile, active ingredients derived from traditional Chinese medicine and natural products, such as curcumin and berberine, have also demonstrated potential value in the field of FAP chemoprevention. Preclinical studies and clinical trials have provided preliminary evidence supporting their efficacy in modulating polyp progression, with distinct advantages in long-term prevention and improving patient outcomes. However, personalized treatment and optimization of drug efficacy will require further basic research and clinical trials.
